# Transcript profiling indicates a widespread role for bacterial-type phosphoenolpyruvate carboxylase in malate-accumulating sink tissues

**DOI:** 10.1093/jxb/erx399

**Published:** 2017-12-12

**Authors:** Michael K Y Ting, Yi-Min She, William C Plaxton

**Affiliations:** 1 Department of Biology, Queen’s University, Kingston, Ontario, Canada; 2 Centre for Biologics Evaluation Biologics and Genetic Therapies Directorate, Health Canada, Ottawa, Ontario, Canada; 3 Department of Biomedical and Molecular Sciences, Queen’s University, Kingston, Ontario, Canada

**Keywords:** Malate, organic acid metabolism, phosphoenolpyruvate carboxylase, RNA-Seq, sink metabolism, tissue-specific gene expression, transcriptomics

## Abstract

Phosphoenolpyruvate carboxylase (PEPC) is an important regulatory enzyme situated at a key branch point of central plant metabolism. Plant genomes encode several plant-type PEPC (PTPC) isozymes, along with a distantly related bacterial-type PEPC (BTPC). BTPC is expressed at high levels in developing castor oil seeds where it tightly interacts with co-expressed PTPC polypeptides to form unusual hetero-octameric Class-2 PEPC complexes that are desensitized to allosteric inhibition by L-malate. Analysis of RNA-Seq and microarray transcriptome datasets revealed two distinct patterns of tissue-specific *BTPC* expression in vascular plants. Species such as *Arabidopsis thaliana*, strawberry, rice, maize, and poplar mainly exhibited pollen- or floral-specific *BTPC* expression. By contrast, *BTPC* transcripts were relatively abundant in developing castor, cotton, and soybean seeds, cassava tubers, as well as immature tomato, cucumber, grape, and avocado fruit. Immunoreactive 118 kDa BTPC polypeptides were detected on immunoblots of cucumber and tomato fruit extracts. Co-immunoprecipitation established that as in castor, BTPCs physically interact with endogenous PTPCs to form Class-2 PEPC complexes in tomato and cucumber fruit. We hypothesize that Class-2 PEPCs simultaneously maintain rapid anaplerotic PEP carboxylation and respiratory CO_2_ refixation in diverse, biosynthetically active sinks that accumulate high malate levels.

## Teaser

Bacterial-type PEP carboxylase is highly expressed in developing sink tissues that accumulate abundant levels of malate.

## Introduction

Phosphoenolpyruvate (PEP) carboxylase (PEPC, EC 4.1.1.31) is a multifaceted and tightly controlled cytosolic enzyme that catalyzes the irreversible β-carboxylation of PEP to form oxaloacetate and P_i_. C_4_ and crassulacean acid metabolism photosynthetic PEPCs have been extensively characterized at the structural and regulatory levels (Izui *et al.*, 2004). However, PEPC plays essential roles in all plant cells, particularly the anaplerotic replenishment of Krebs’ cycle intermediates withdrawn for biosynthesis and N-assimilation (O’Leary *et al.*, 2011*b*). Most plant PEPCs studied to date exist as Class-1 PEPC homotetramers composed of identical plant-type PEPC (PTPC) subunits of ~105–110 kDa (O’Leary *et al.*, 2011*b*). The activity of Class-1 PEPCs is: (i) potently modulated by various allosteric effectors, particularly the feedback inhibitor L-malate, and (ii) subject to reciprocal control by reversible phosphorylation, for activation, and monoubiquitination, for inhibition, at highly conserved serine and lysine residues, respectively (Uhrig *et al.*, 2008*b*; O’Leary *et al.*, 2011*b*; Shane *et al.*, 2013; Ruiz-Ballesta *et al*., 2014; Figueroa *et al.*, 2016). In 2003 and 2004, interrogation of *Arabidopsis thaliana,* rice (*Oryza sativa*), and soybean (*Glycine max*) *PEPC* gene families led to the unexpected discovery that, alongside their various *PTPC* genes, all three genomes also encode and express a distantly related and enigmatic *bacterial-type PEPC* (*BTPC*) gene whose deduced amino acid sequence shares higher similarity with PEPCs from eubacteria than with PTPCs (Sánchez and Cejudo, 2003; Sullivan *et al.*, 2004). These novel PEPCs were thus referred to as BTPCs, whereas all remaining C_3_, C_4_, and crassulacean acid metabolism PEPCs were classified as PTPCs. All plant genomes sequenced to date, including that of ancestral green algae, contain at least one *BTPC* gene. The BTPCs constitute a monophyletic group, separate from the PTPCs, eubacterial, and archaeal PEPCs and appear to have evolved in green algae (O’Leary *et al.*, 2011*b*)

Insights into BTPC function arose when novel, high molecular mass Class-2 PEPC heteromeric complexes composed of tightly associated PTPC and BTPC subunits were purified from green microalgae and then from developing castor oil seeds (COS; *Ricinus communis*) (Rivoal *et al.*, 1998; Rivoal *et al.*, 2001; Blonde and Plaxton, 2003; Tripodi *et al.*, 2005; Gennidakis *et al.*, 2007; Uhrig *et al.*, 2008*a*). Tryptic peptide sequencing by LC MS/MS identified RcPPC3 and RcPPC4, namely castor PTPC and BTPC, respectively, as the subunits of COS Class-2 PEPC (Blonde and Plaxton 2003, Gennidakis *et al.*, 2007). *BTPC* and *PTPC* transcripts and polypeptides are coordinately expressed during COS development; their developmental profiles correlate with Class-2 PEPC formation as well as with the stages of rapid endosperm growth and oil accumulation (Blonde and Plaxton, 2003; Gennidakis *et al.*, 2007; O’Leary *et al.*, 2011*a*; Ying *et al.*, 2017). Although both subunit types exhibit PEPC activity within the Class-2 PEPC complex, the BTPC subunits demonstrated remarkable insensitivity towards PTPC allosteric inhibitors such as L-malate (O’Leary *et al.*, 2009; O’Leary *et al.*, 2011*b*). BTPC also functions as a regulatory subunit by significantly reducing the allosteric effector sensitivity of associated PTPC subunits within Class-2 PEPC (Blonde and Plaxton, 2003; O’Leary *et al.*, 2009). Furthermore, COS Class-2 PEPC localizes to the outer mitochondrial envelope, an interaction mediated by its BTPC subunits *in vivo* (Park *et al.*, 2012). It is also notable that exogenous malate supported maximal rates of fatty acid synthesis by purified leucoplasts from developing COS (Smith *et al.*, 1992), and that malate import from the cytosol into the COS leucoplast stroma is mediated by a malate/Pi translocator within the leucoplast envelope (Eastmond *et al.*, 1997). The oxidation of malate into acetyl-CoA by plastid isozymes of NADP-malic enzyme and the pyruvate dehydrogenase complex generates all the acetyl-CoA and NAD(P)H, needed for long chain fatty acid synthesis. The collective results support the hypothesis that the BTPC and PTPC containing Class-2 PEPC complex rapidly refixes respired CO_2_ while simultaneously facilitating a large anaplerotic flux to replenish malate required for the abundant fatty acid synthesis that dominates the carbon metabolism of developing COS (Blonde and Plaxton, 2003; O’Leary *et al.*, 2009; O’Leary *et al.*, 2011a, b; Park *et al.*, 2012). Significant *BTPC* and *PTPC* expression also occurs in immature peanut (*Arachis hypogaea*) embryos (Yu *et al.*, 2010; Pan *et al*., 2013), indicating that Class-2 PEPCs might contribute to anaplerotic photosynthate partitioning at the PEP branch point in a variety of developing oilseeds. This could be particularly advantageous in bulky, non-photosynthetic oilseeds such as COS or peanuts that likely have limited capacities for atmospheric gas exchange. However, further development of this hypothesis has been hampered because evidence for Class-2 PEPC complexes and/or BTPC polypeptides in seeds has thus far been restricted to developing COS (Blonde and Plaxton, 2003; Tripodi *et al.*, 2005; Uhrig *et al.*, 2008*a*; O’Leary *et al.*, 2009; O’Leary *et al.*, 2011*b*).

The aim of the current study was to characterize vascular plant *BTPC* expression patterns by analysing publicly available RNA-Seq and microarray transcriptomic datasets. Several sink tissues exhibiting abundant levels of *BTPC* transcripts were shown to contain a Class-2 PEPC complex composed of associated PTPC and BTPC subunits. The results provide surprising and important insights into the expression and potential metabolic roles of vascular plant BTPC and Class-2 PEPCs.

## Materials and methods

### Plant material

Castor *(Ricinus communis,* cv. Baker 296), tomato (*Solanum lycopersicum,* cv. Beefsteak), and cucumber (*Cucumis sativus*, cv. Marketmore) plants were cultivated in a greenhouse at 24°C and 60% humidity under natural light supplemented with 16 h of artificial light. All tissues were rapidly harvested, snap frozen in liquid N_2_, and stored at -80°C until used.

### Identification and phylogenetic analysis of *BTPC* genes

The amino acid sequence of castor BTPC (RcPPC4; Genbank ID: EF634318.1) was subjected to BLAST in NCBI, Phytozome v12.0, and Genoscope databases to identify BTPC orthologs (see Supplementary Dataset S1 at *JXB* online). The genomic sequence of avocado (*Persea americana*) *BTPC* (contig ID: PA10006483) was derived from the supplementary data of Kilaru and co-workers (2015) and its amino acid sequence deduced using GENSCAN (http://genes.mit.edu/GENSCAN.html). Amino acid sequences were aligned using muscle (default settings, http://www.drive5.com/muscle) and phylogenetic analysis conducted using MEGA7 (Kumar *et al.*, 2016). The evolutionary history was inferred using the Maximum Likelihood method based on the Le Gascuel model (Le and Gascuel, 2008). A discrete Gamma distribution, with five categories, was used to model evolutionary rate differences among sites. Bootstrap analysis was carried out with 100 replicates.

### Analysis of BTPC expression from RNA-Seq and microarray transcriptome datasets

Profiling of *BTPC* transcripts was restricted to publicly available RNA-Seq and microarray datasets for sequenced plant genomes having robust gene annotations. The first round of data mining employed the Bio-Analytic Resource for Plant Biology (BAR) database (www.BAR.utoronto.ca). The gene identifier for Arabidopsis *BTPC* (At1g68750; *AtPPC4*) was used as the search query in the BAR ‘expressolog tree’ to locate BTPC orthologs and *BTPC* expression data derived from the respective eFP browsers (Winter *et al.*, 2007). RNA-Seq data for strawberry (*Fragaria vesca*) was obtained from the strawberry eFP browser (http://mb3.towson.edu/efp/cgi-bin/efpWeb.cgi) (Hawkins *et al.*, 2017). The Sequence Read Archive (https://www.ncbi.nlm.nih.gov/sra) was also interrogated for available RNA-Seq studies for each species. Plants with abundant, tissue-specific transcriptomic data were further analysed. If authors of the RNA-Seq publications included annotated transcript data with their supplementary information, the reads per kilobase of gene per million reads (RPKM) or fragments per kilobase of transcript per million reads (FPKM) for *BTPC* were taken directly from these files. If no annotated data were available, the raw reads were downloaded from the Sequence Read Archive and datasets aligned to the reference genomes, obtained from https://phytozome.jgi.doe.gov/pz/portal.html, using Bowtie version 1.1.2 (http://bowtie-bio.sourceforge.net/index.shtml). Alignments were done using restricted parameters that only reported unique single alignments. Sequence Alignment Map format files were converted into Binary Alignment Map format using ‘SAMtools’ (Li *et al.*, 2009) and then transformed into Browser Extensible Data format using bedtools (Quinlan and Hall, 2010). The Browser Extensible Data allowed the number of mapped reads corresponding to *BTPC* genes to be normalized to RPKM using a custom Perl script (Supplementary File S1). If replicate determinations were available for the mined data, the average values were calculated and reported as means±SEM (Supplemental Dataset S2). Expression maps (heat maps) were generated using heatmap.2 software (https://cran.r-project.org/package=gplots).

### Protein extraction

Quick frozen COS endosperm was ground to a powder under liquid N_2_ and homogenized (1:2; w/v) in 50 mM HEPES-KOH at pH 7.5 containing 1 mM EDTA, 1 mM EGTA, 25 mM NaF, 1 mM Na_3_VO_4_, 1 mM Na_2_MoO_4_, 0.1% (v/v) Triton X-100, 20% (v/v) glycerol, 10 mM MgCl_2_, 1% (w/v) poly(vinylpolypyrrolidone), 2 mM phenylmethylsulphonyl fluoride, 2 mM 2,2’-dipyridyl disulphide, and 5 µl ml^-1^ ProteCEASE-100 (G-Biosciences). Tomato and cucumber fruit homogenates were prepared the same way except that the extraction buffer contained 500 mM TRIS-HCl at pH 7.5, instead of 50 mM HEPES-KOH, and was supplemented with 5 mM NaHCO_3_ and 10 mM L-ascorbic acid. All homogenates were centrifuged at 4°C and 15000 *g* for 10 min and the supernatants filtered through two layers of Miracloth. Clarified extracts were immediately boiled in SDS-PAGE sample buffer for 3 min or frozen in liquid N_2_ and stored at -80°C for future use.

### Electrophoresis, immunoblotting, and in-gel PEPC activity staining

SDS and non-denaturing PAGE using a Bio-Rad Protean III mini-gel apparatus, immunoblotting, and in-gel PEPC activity staining were performed as described by Blonde and Plaxton (2003). Anti-COS PTPC/RcPPC3 and anti-COS BTPC/RcPPC4 immune sera, anti-PTPC and anti-BTPC, respectively, were raised in rabbits as previously described (Gennidakis *et al.*, 2007; O’Leary *et al.*, 2009). Antigenic polypeptides were visualized using a peroxidase-conjugated α-rabbit secondary antibody (Sigma) with Clarity^TM^ Western ECL Blotting Substrate (Bio-Rad) and imaged using a Bio-Rad ChemiDoc Touch Imaging System. All immunoblots were replicated at least three times with representative results shown in the figures.

### Co-immunoprecipitation

Anti-PTPC IgG (Gennidakis *et al.*, 2007) was purified from the corresponding immune serum using Pierce Protein A Chromatography Cartridges (Thermo Fisher Scientific) according to the manufacturer’s instructions. Purified IgG was coupled to Surebeads^TM^ Protein-G Magnetic Beads (Bio-Rad) followed by a 1 h incubation at 24°C with clarified protein extracts from developing cucumber and immature (green) tomato fruit. After thorough washing, bound proteins were eluted with 20 mM glycine-HCl at pH 2.0 and rapidly neutralized by the addition of unbuffered TRIS. Co-immunopurification (co-IP) eluates were subjected to SDS-PAGE and anti-PTPC and anti-BTPC immunoblotting as described above.

### Mass spectrometry

Coomassie Blue-stained bands corresponding to 118 kDa BTPC and 107 kDa PTPC polypeptides were excised from SDS gels of the tomato fruit co-IP eluate, digested by sequencing grade trypsin (Promega, Madison, WI), and tryptic peptides extracted and lyophilized as described previously (Uhrig *et al.*, 2008*b*). Digests were reconstituted in 10 µL of 0.2% (v/v) formic acid and analysed by Thermo Scientific Orbitrap Fusion coupled with an Easy-nLC 1000 system. The peptides were trapped at a flow rate of 5 µL min^-1^ of solvent A (0.1% formic acid in water) on an Acclaim PepMap 100 C18 column (75 µm internal diameter, 2 cm in length, and 3 µm particle diameter), followed by separation with an Easy-spray PepMapTM RSLC C18 column (75 µm internal diameter, 15 cm in length, and 3 µm particle diameter). A linear gradient from 5 to 30% of solvent B (0.1% formic acid in acetonitrile) over 60 min, 85% of solvent B for another 10 min, and then 98% of solvent A at 400 nL min^-1^ was used for peptide binding and elution, analytical column washing, and equilibration. Online LC MS/MS analysis was performed using the data dependent top-N MS/MS scans. The initial MS survey scans were acquired by Orbitrap at the solving power of 120,000 from *m/z* 350 to 1600, and subsequent low-energy collision-induced dissociation of the peptides by ion-trap were selectively fragmented at precursor ions with multiply charged states using 30% normalized collision energy. The AGC target value was set up for MS at 4.0e5 and MS/MS at 5.0e4. Dynamic exclusion was enabled for a period of 30 s. Raw data from the LC MS/MS analyses were separately converted into MGF files using Proteome Discoverer 2.1 software (ThermoFisher Scientific Inc.) and then searched against the NCBI databases for Viridiplantae (green plants, 4,240,684 sequences downloaded on August 30, 2016) using MASCOT (Matrix Science, London, UK). Search parameters were restricted to tryptic peptides at a maximum of two missed cleavages. Cysteine carbamidomethylation was designated as a fixed modification and phosphorylation of serine/threonine/tyrosine, deamidation of asparagine and glutamine, and oxidation of methionine were considered variable modifications. Phosphorylation sites were validated by manual inspection of MS/MS spectra with predicted fragments. Mass tolerances were set up to 10 ppm for the Orbitrap-MS ions and 1 Da for ion trap MS/MS fragment ions. Peptide assignments and reliable protein identification were based on the ion scores for MS/MS matches at the default significance threshold of *P*<0.05.

### Partial purification of PEPC from immature tomato fruit

Proteins were extracted from 41 g of immature (green) tomato fruit at 14 days post-anthesis (DPA) as described above. The clarified extract was brought up to 20% ammonium sulfate (saturation) and stirred at 4°C for 15 min. After centrifugation at 30000 *g* for 15 min the supernatant fluid was loaded at 1.5 ml min^-1^ on to a column (1.5 cm × 2.8 cm) of Butyl Sepharose 4 Fast Flow (GE Healthcare) pre-equilibrated with 50 mM TRIS-HCl at pH 7.6 containing 1 mM EDTA, 1 mM EGTA, 5 mM MgSO_4_, 5 mM NaHCO_3_, 5 mM malate, and 20% (NH_4_)_2_SO_4_ (saturation). After the *A*_*280*_ approached baseline, PEPC activity was eluted using the same buffer, except that it lacked (NH_4_)_2_SO_4_ and contained 10% (v/v) ethylene glycol. Eluted proteins were concentrated to about 10 mg ml^-1^ using an Amicon Ultra-15 centrifugal filter unit (100 kDa MWCO; EMD Millipore).

In this article the terms ‘malate’ and ‘citrate’, which represent the conjugate base of malic and citric acids, refer to all physiological forms of each compound.

## Results and discussion

### Identification and phylogenetic analysis of plant BTPC genes

**Fig. 1.**
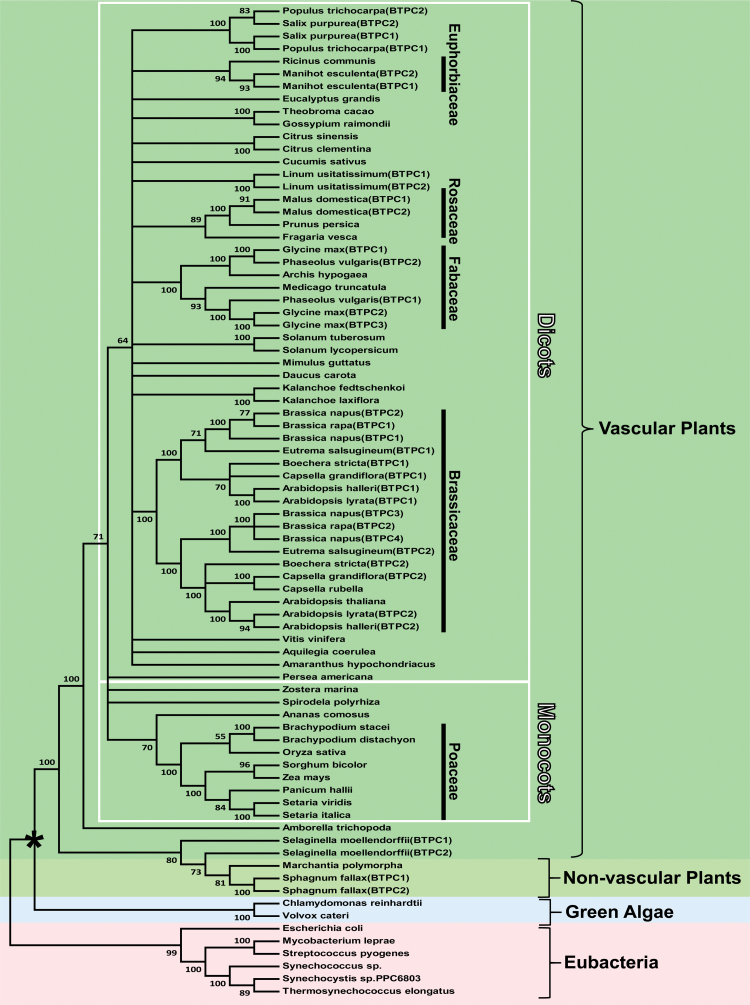
Phylogenetic analysis of plant BTPCs and several eubacterial PEPCs. The maximum likelihood tree was constructed using deduced amino acid sequences for the various PEPCs (gene identifiers available in Supplementary Table S1) from a muscle alignment conducted using MEGA7 (Kumar *et al.*, 2016). Bootstrap analysis was carried out with 100 replicates and the numbers at the branch points correspond to percentage bootstrap frequencies. Bootstrap values less than 50 were collapsed. The tree was rooted using eubacterial PEPC as the outgroup. The asterisk denotes the divergence of plants from green algae. Gene identifier numbers for the various PEPCs are listed in Supplementary Dataset S1.

**Fig. 2.**
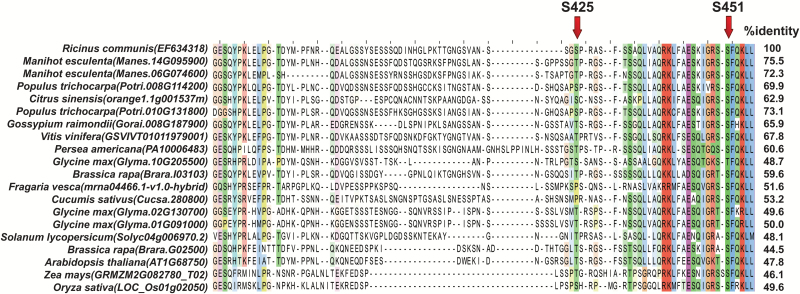
Alignment of the intrinsically disordered region of deduced plant BTPC polypeptides. The arrows indicate experimentally verified *in vivo*, inhibitory phosphorylation sites of COS BTPC, namely Ser-425 and Ser-451 (O’Leary *et al.*, 2011*c*; Dalziel *et al.*, 2012). Amino acid residues are colored according to their degree of conservation based upon Clustal×Color Scheme (http://www.jalview.org/help/html/colourSchemes/clustal.html).

Although many plant genomes contain a single *BTPC* gene, species such as field mustard (*Brassica rapa),* soybean (*Glycine max*), cassava (*Manihot esculenta*), and poplar (*Populus trichocarpa*) appear to contain two or more *BTPC* genes (Fig. 1 and Supplementary Dataset S1). *BTPC* genes are characterized by about 20 exons, whereas *PTPC* genes contain approximately 10 exons, irrespective of whether they originate from C_3_, C_4_, or crassulacean acid metabolism plants (Sánchez and Cejudo, 2003; O’Leary *et al.*, 2011*b*). These differences support the hypothesis that *PTPC* and *BTPC* evolved independently from a common ancestral *PEPC* gene very early during the evolution of the plant kingdom. Phylogenetic analysis of deduced BTPC polypeptides was consistent with the divergence of green algae and vascular plants, as well as monocots and dicots (Fig. 1). All vascular plant BTPC polypeptides ranged from 114–120 kDa and contained the characteristic domains and residues important for PEPC structure and catalysis (Izui *et al.*, 2004; O’Leary *et al.*, 2011*b*), while exhibiting 77–92% sequence identity with castor BTPC (Supplementary Fig. S1). They were further classified as BTPCs according to three BTPC-specific criteria: (i) presence of the C-terminal tetrapeptide (R/K)NTG representative of non-archaeal, prokaryotic-like PEPCs, (ii) absence of the N-terminal seryl phosphorylation motif characteristic of PTPCs, namely acid-base-XX**pS**IDAQLR, and (iii) presence of an approximate 10 kDa intrinsically disordered region corresponding to residues 325–467 of castor BTPC (Supplementary Fig. S1 and Fig. 2) (O’Leary *et al.*, 2011*b*).

As previously noted (Park *et al.*, 2012), sequence variation between the different BTPCs mainly occurs within their disordered region, whose sequence identity with the disordered region of castor BTPC ranges from about 44–75% (Fig. 2). Intrinsically disordered regions typically provide a docking site to promote protein–protein interactions by recruiting binding partners (Dunker *et al.*, 2005). As documented for other disordered region-containing proteins (Brown *et al.*, 2002), the disordered region of BTPC exhibits a higher mutation rate relative to corresponding ordered domains and may be a driving force for evolution to achieve optimal binding efficiency. BTPC is no exception since its disordered region mediates a tight interaction with co-expressed PTPC subunits to form the high molecular mass Class-2 PEPC complex of developing COS (Park *et al.*, 2012). It is also notable that the *in vivo* regulatory Ser-451 phosphorylation site of the disordered region of COS BTPC (Dalziel *et al.*, 2012), as well as several Ser-451 flanking residues, are conserved in all deduced BTPC polypeptides, with the exception of soybean BTPC-1 (Glyma.10G205500) in which the Ser at this position is substituted by a Thr (Fig. 2). RcCDPK1 was recently identified as a calcium dependent protein kinase isozyme that catalyzes *in vivo* regulatory inhibitory BTPC phosphorylation at Ser-451 in developing COS (Hill *et al.*, 2014; Ying *et al.*, 2017). BTPC characterization from additional species will therefore help to corroborate and extend the role of calcium-dependent BTPC phosphorylation. In addition the proline-directed Ser-425 regulatory phosphorylation site of , COS BTPC (O’Leary *et al.*, 2011*c*) is also present in the disordered region of several BTPC orthologs aligned in Fig. 2, supporting the hypothesis that multiple levels of post-translational BTPC control occurs throughout the plant kingdom.

### Transcriptomic analysis reveals two distinct patterns of BTPC expression in vascular plants

**Fig. 3.**
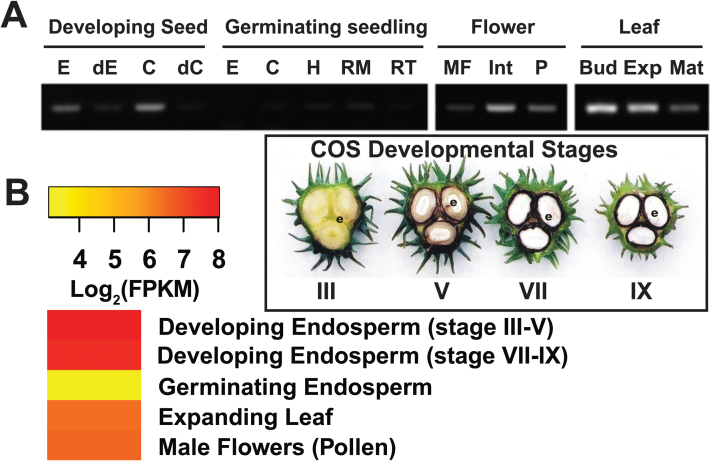
Correlation of castor *BTPC* expression. (A) Castor *BTPC* expression profiles as determined via RT-PCR (reproduced from O’Leary *et al.*, 2011*a*. Tissue-specific expression and post-translational modifications of plant- and bacterial-type phosphoenolpyruvate carboxylase isozymes of the castor oil plant, *Ricinus communis* L. Journal of Experimental Botany 62, 5485–5495). E, endosperm; C, cotyledon; dC and dE, cotyledon and endosperm, respectively, from stage VII developing COS that had been depodded for 72 h; H, hypocotyl; RM, root middle; RT, root tip; MF, male flower; Int, integument; P, pericarp; Bud, leaf bud; Exp., expanding leaf; Mat., mature leaf. (B) Expression map (heat map) of castor *BTPC* expression derived from RNA-Seq data of Brown and co-workers (2012). Stages III, V, VII, and IX respectively correspond to the heart-shaped embryo, mid-cotyledon, full cotyledon, and maturation stages of COS development.

**Fig. 4.**
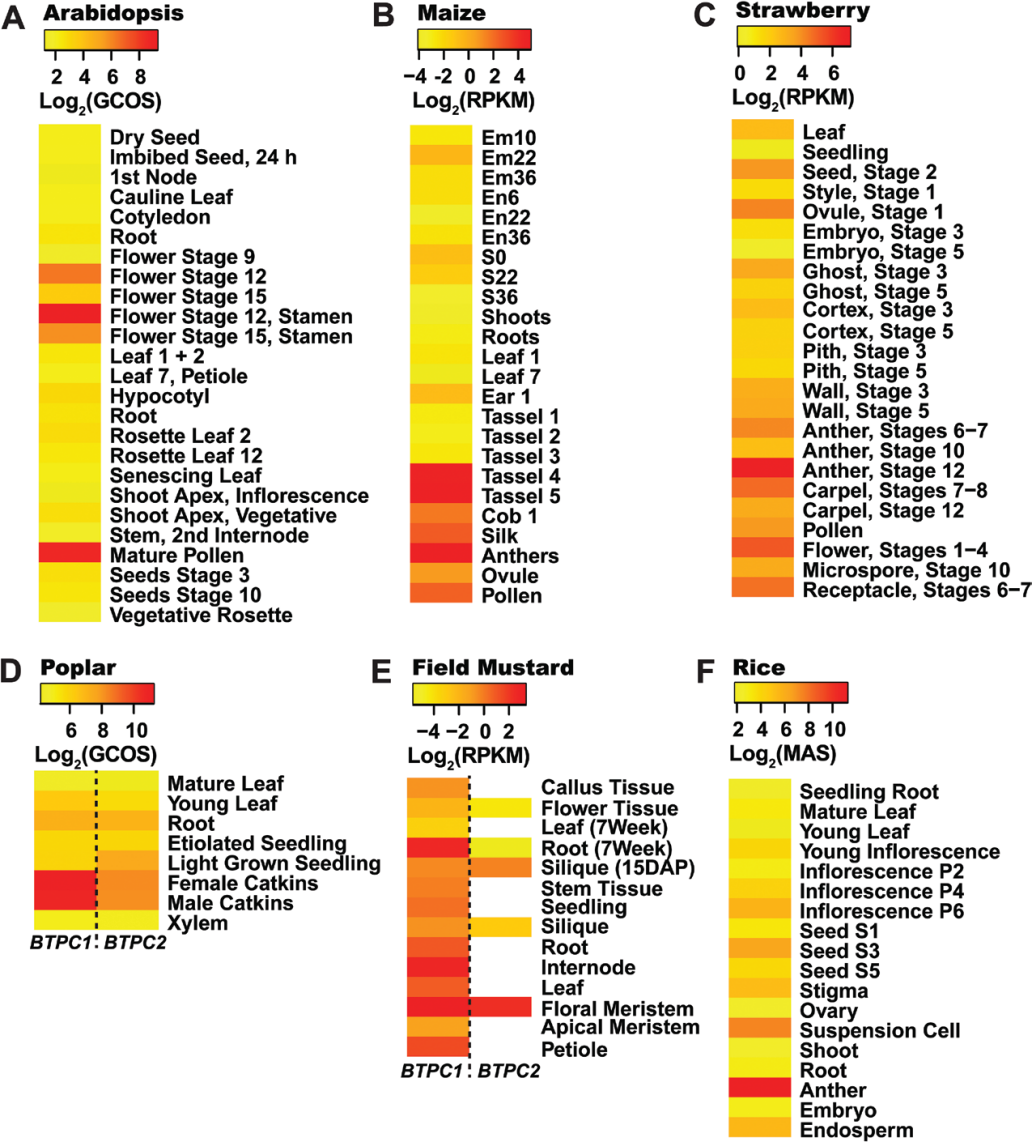
Expression maps (heat maps) of species that mainly exhibit pollen/floral-specific *BTPC* expression. The expression values for each species were Log_2_-transformed and scaled accordingly to contrast tissues within a particular species that exhibit the lowest and most abundant *BTPC* transcript levels. Empty (white) rectangles denote tissues with undetectable *BTPC* transcripts. Details of transcriptomic data are available in Supplementary Dataset S2. RMA, Robust Microarray Average values; GCOS, Normalized GeneChip Operating Software values; MAS, Normalized MAS 5.0 Software values.

**Fig. 5.**
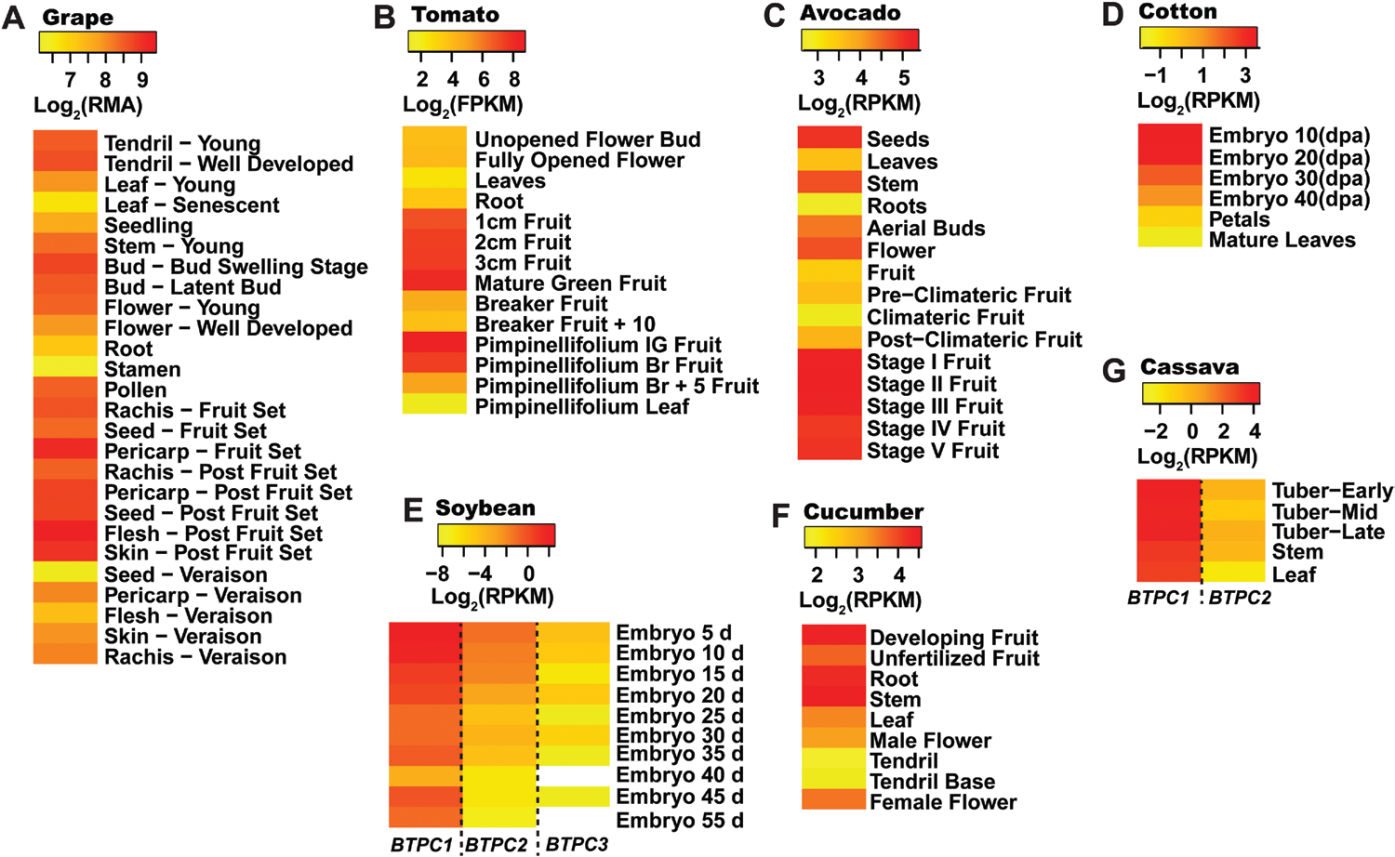
Expression maps (heat maps) of plants exhibiting significant *BTPC* expression in non-floral, developing sink tissues. The expression values for each species were Log_2_-transformed and scaled accordingly to contrast tissues within a particular species that exhibit the lowest and most abundant *BTPC* transcript levels. Empty (white) rectangles denote tissues with undetectable *BTPC* transcripts. Details of transcriptomic data are available in Supplementary Dataset S2. RMA, Robust Microarray Average values; GCOS, Normalized GeneChip Operating Software values; MAS, Normalized MAS 5.0 Software values.

Fourteen species were selected for transcriptomic analysis based upon availability of their annotated genome sequences, as well as corresponding RNA-Seq and/or microarray transcriptomic datasets for tissues of interest, namely developing fruits or seeds, leaves, pollen, and roots (Supplementary Dataset S2). In particular, RNA-Seq is a remarkable sequencing technology that provides accurate, high-throughput and genome-wide quantification of transcription. *BTPC* expression values discussed below and reported in Figs 3–5 and Supplementary Dataset S2 were derived from a range of transcriptome profiling approaches and are thus not directly comparable across the fourteen species that we analysed. However, our intent was to identify tissues within a particular species in which *BTPC* transcripts are most abundantly expressed, thereby providing insights regarding the potential tissue-specific expression of BTPC polypeptides and Class-2 PEPC complexes. Transcriptome profiling for tissues of several species that we analyzed had been reported for two (strawberry) or three (Arabidopsis, grape, rice, and poplar) biological replicates; corresponding mean±SEM *BTPC* transcript levels were therefore calculated and demonstrate that tissue-specific *BTPC* expression patterns in these species were quite reproducible (Supplementary Dataset S2). *BTPC* expression was normalized relative to a reference gene for nine of the 14 species that we analysed and highly similar tissue-specific *BTPC* expression patterns were observed as compared with those based upon absolute quantification of *BTPC* transcripts (Supplementary Dataset S2). To further validate our comparative analysis of *BTPC* expression, RNA-Seq data for castor and microarray data for Arabidopsis were correlated with the respective *BTPC* mRNA profiles previously obtained via RT-PCR using *BTPC* gene-specific primers. The respective results consistently agree that *BTPC* transcripts are abundant during the early stages of COS development and absent in germinating COS (Fig. 3) (O’Leary *et al.*, 2011*a*; Brown *et al.*, 2012), but are low or undetectable in most Arabidopsis tissues except for pollen (Fig. 4) (Sánchez and Cejudo 2003; Schmid *et al.*, 2005, Igawa *et al.*, 2010). As discussed below, plant *BTPC* expression appears to follow one of these two restricted patterns; namely species such as Arabidopsis in which *BTPC* mainly exhibits pollen- or anther/floral-specific expression, as opposed to species like castor that exhibit maximal *BTPC* expression in certain biosynthetically active, non-floral sink tissues, namely developing seeds, fruits, or tubers (Figs 3–5).

Monocot and dicot species that mainly exhibited pollen- or anther/floral-specific *BTPC* expression appear to be relatively common since Arabidopsis, field mustard, rice, poplar, strawberry, maize (*Zea mays*), and lily (*Lilium longiflorum*) all fall under this category (Fig. 4). Interestingly, all three monocots that were analysed, namely rice, lily, and maize, demonstrated pollen-specific *BTPC* expression, suggesting an evolutionary role in pollen development prior to the monocot-dicot divergence. Field mustard *BTPC1* shared a similar expression profile to Arabidopsis *BTPC*, being generally low except for the flowers and roots (Fig. 4). This was not surprising considering that both species belong to the crucifer family. However, the field mustard genome contains two *BTPC* genes, the first of which is more widely expressed (Fig. 4); this may signify alternative BTPC functions within this species. Results of Fig. 4 support the hypothesis of Igawa and co-workers (2010) that BTPC/Class-2 PEPC may promote enhanced lipid and protein synthesis in developing pollen.

By contrast, *BTPC* transcripts were relatively abundant in developing seeds of castor, avocado, soybean, cotton (*Gossypium raimondii*), and grape (*Vitis vinifera*) (Figs 3 and 5). Although developing soybeans generally exhibited low *BTPC1-3* transcript levels, enhanced *BTPC1* expression occurred during the earliest stage of soybean embryo development (Fig. 5). Prominent *BTPC* expression also occurred in immature fruits of cucumber, tomato, avocado, and grape, as well as developing cassava tubers (Fig. 5).

### BTPC polypeptides of immature cucumber and tomato fruit interact with co-expressed PTPC polypeptides to form Class-2 PEPC complexes

**Fig. 6.**
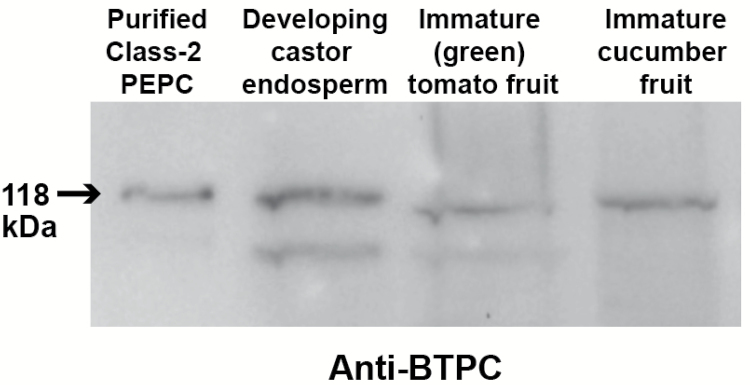
Immunoblot analysis of BTPC polypeptides in extracts prepared from developing castor seeds, and immature cucumber and tomato fruit. Purified recombinant chimeric Class-2 PEPC (50 ng) (O’Leary *et al.*, 2009) consisting of 1:1 ratio of 118 kDa RcPPC4 (castor BTPC) and 107 kDa AtPPC3 (an Arabidopsis PTPC isozyme) polypeptides was used as a positive control (lane 1). Protein extracts were prepared from stage V developing COS endosperm (lane 2), immature green tomato fruit (lane 3), and developing cucumber fruit (lane 4). Samples were subjected to SDS-PAGE, with 50 µg protein used per lane^,^ and immunoblotting using anti-BTPC. Antigenic polypeptides were visualized using a peroxidase-conjugated secondary antibody and enhanced chemiluminescent detection.

Previous research with the castor oil plant demonstrated that its restricted pattern of tissue-specific *BTPC* expression is paralleled by the accumulation of high molecular mass Class-2 PEPC complexes composed of an equivalent ratio of 118 kDa BTPC and 107 kDa PTPC polypeptides (Blonde and Plaxton, 2003, Gennidakis *et al.*, 2007, Uhrig *et al.*, 2008*a*, O’Leary *et al.*, 2011*a*). Since tomato and cucumber fruit have considerable agronomic importance and were easy for us to obtain and grow, they were selected to establish if their *BTPC* transcripts are translated into BTPC polypeptides, and if so, whether this also leads to formation of Class-2 PEPC complexes via BTPCs interaction with co-expressed PTPC polypeptides. Clarified extracts from immature (green) tomato and cucumber fruit were initially subjected to SDS-PAGE and immunoblotting using anti-BTPC. As seen in Fig. 6, 118 kDa anti-BTPC immunoreactive polypeptides that are similar to the predicted sizes of the deduced tomato and cucumber BTPC polypeptides (Supplementary Fig. S1) were observed that co-migrated with castor BTPC. The tomato results corroborate a recent proteomic study which detected significant levels of BTPC peptides in tryptic digests of protein extracts prepared from unripe (green) tomato fruit (Szymanski *et al.*, 2017). The less intense, lower molecular mass anti-BTPC immunoreactive polypeptide present on the immunoblots of the castor and tomato extracts is probably an artefact arising from limited *in vitro* proteolysis of the 118 kDa BTPC polypeptide by endogenous proteases (Gennidakis *et al*., 2007).

**Fig. 7.**
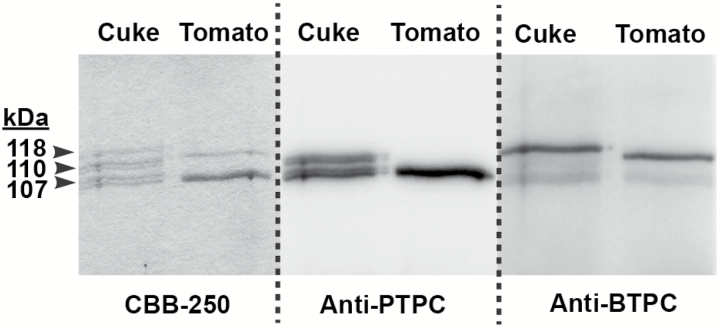
Co-immunoprecipitation of BTPC and PTPC polypeptides from immature cucumber and tomato fruit. Co-IP was performed using purified anti-PTPC IgG as described in the Materials and Methods. Co-IP eluates were subjected to SDS-PAGE followed by Coomassie Blue R-250 (CBB-250) staining and immunoblotting with anti-PTPC or anti-BTPC as indicated. ‘Cuke’, cucumber.

To determine whether tomato and cucumber BTPCs also form Class-2 PEPC complexes with endogenous PTPC polypeptides, co-IP using anti-PTPC was performed on protein extracts followed by SDS-PAGE and anti-BTPC and anti-PTPC immunoblotting of the co-IP eluates (Fig. 7). Owing to their low (~40%) sequence identity PTPC and BTPC polypeptides are immunologically distinct (Blonde and Plaxton, 2003; Gennidakis *et al.*, 2007; Uhrig *et al.*, 2008*a*; O’Leary *et al.*, 2009; Igawa *et al.*, 2010). The results confirmed that a tight physical interaction occurs between tomato or cucumber BTPC and the corresponding PTPC since 118 kDa BTPC polypeptides effectively co-immunopurified with 107 kDa PTPC polypeptides from both tissues (Fig. 7). LC MS/MS of tryptic digests revealed that the 107 and 118 kDa protein-staining polypeptides observed following SDS-PAGE of the tomato fruit anti-PTPC co-IP eluate (Fig. 7) contained single proteins that were respectively identified as a tomato fruit-specific PTPC isozyme (gi|1003700994; sequence coverage=79%; Mascot score=1422) (Guillet *et al.*, 2002) and tomato BTPC (gi|460382446; sequence coverage=34%; Mascot score=182). Furthermore, LC MS/MS also established that the co-immunoprecipitated 107 kDa PTPC polypeptide was phosphorylated at Ser-11 (Supplementary Fig. S2), supporting the hypothesis of Guillet and co-workers (2002) that the fruit-specific PTPC of developing tomatoes is activated by *in vivo* phosphorylation at Ser-11. This site occurs within the well-known and much studied N-terminal seryl-phosphorylation motif characteristic of all known PTPCs (O’Leary *et al.*, 2011*b*).

**Fig. 8.**
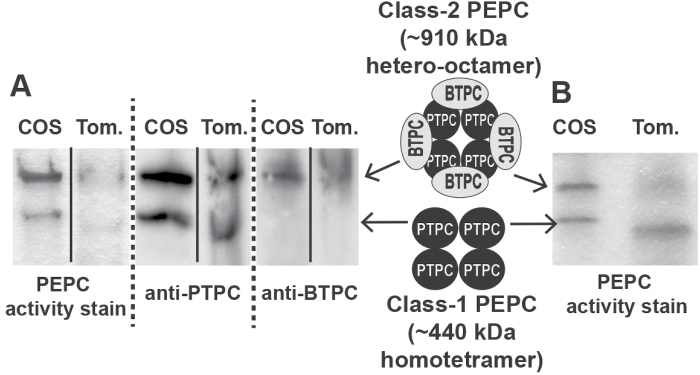
Non-denaturing PAGE of Class-1 and Class-2 PEPCs from developing castor beans and tomato fruit. (A) In-gel PEPC activity staining and anti-PTPC and anti-BTPC immunoblotting was performed on clarified extracts prepared from stage V developing castor oilseeds (COS) endosperm and immature (green) tomatoes. (B) Non-denaturing PAGE followed by PEPC in-gel activity staining was performed on a clarified extract prepared from stage V developing COS endosperm and Butyl-Sepharose purified PEPC from immature (green) tomato fruit. Butyl-Sepharose FPLC was performed to improve resolution of tomato Class-1 PEPC versus Class-2 PEPC activity staining bands. Tom., tomato.

We next assessed the subunit composition and approximate native molecular mass of Class-1 and Class-2 PEPCs from immature tomato fruit by non-denaturing PAGE of clarified extracts followed by in-gel PEPC activity staining and immunoblotting using anti-BTPC and anti-PTPC. A pair of PEPC activity-stained and anti-PTPC immunoreactive bands were observed that respectively co-migrated with the previously characterized 440 kDa Class-1 PEPC homotetramer and 910 kDa Class-2 PEPC hetero-octamer from developing COS (Fig. 8) (Blonde and Plaxton, 2003; Tripodi *et al.*, 2005; Gennidakis *et al.*, 2007; Uhrig *et al.*, 2008*a*; O’Leary *et al.*, 2009, O’Leary *et al.*, 2011a, c). As indicated by the co-IP and LC MS/MS results discussed above, the PTPC subunits of the tomato Class-1 and Class-2 PEPCs originate from a fruit-specific *PTPC* gene (Guillet *et al.*, 2002). Analogous results were previously reported for developing castor beans in which association of an identical PTPC subunit, RcPPC3, with unrelated BTPC, RcPPC4, polypeptides led to marked physical and kinetic differences between Class-1 and Class-2 PEPCs isolated from this tissue, which oligomerize as PTPC homotetramers and PTPC-BTPC hetero-octamers, respectively (Blonde and Plaxton, 2003, Gennidakis *et al.*, 2007). The tomato results are particularly notable since tomato has become the primary experimental model for molecular and biochemical studies of fleshy fruit development and ripening.

### Significant BTPC expression occurs in sink tissues that accumulate high concentrations of malate

An important and distinctive kinetic feature of green algal and vascular plant, namely COS, Class-2 PEPCs is that their constituent PTPC and BTPC subunits are highly desensitized to allosteric effectors, particularly the feedback inhibitor malate, relative to most PTPC-containing Class-1 PEPCs (Rivoal *et al.*, 1998; Rivoal *et al.*, 2001; Blonde and Plaxton, 2003; O’Leary *et al.*, 2009). For example, the phosphorylated and dephosphorylated forms of Class-1 PEPC from developing COS exhibited very low *I*_*50*_(malate) values of 0.075 and 0.03 mM, respectively (Tripodi *et al.*, 2005); whereas the *I*_*50*_(malate) values of the PTPC and BTPC subunits of COS Class-2 PEPC were about 1.6 and 11 mM, respectively (Blonde and Plaxton, 2003; O’Leary *et al.*, 2009). Thus, Class-2 PEPCs have been hypothesized to maintain rapid anaplerotic PEP carboxylation under physiological conditions, namely elevated cytosolic malate levels, which would potently inhibit the corresponding Class-1 PEPC. Malate is partitioned between the cytosol and vacuole in plant cells (Martinoia and Rentsch, 1994). Its cytosolic concentration has been determined for several plant tissues; for example ranging from about 1–2.5 mM in spinach leaves, 4–8 mM in maize root tips, and 15–20 mM in sycamore suspension cells (Gout *et al.*, 1993; Martinoia and Rentsch, 1994). The total intracellular malate concentration of developing COS endosperm was recently estimated to be as high as 80 mM (Table 1) (Wheeler *et al.*, 2016), so it is reasonable to assume that cytosolic malate levels in excess of several mM exist within this tissue.

The results demonstrate that significant non-floral *BTPC* expression is not unique to developing COS but also occurs in diverse sink tissues of other species that also accumulate significant levels of malate (Fig. 5 and Table 1). For example, grape berries contain up to 150 mM malate, which reaches maximal levels about a week before ripening (Table 1) (Diakou *et al.*, 2000); *BTPC* transcripts also peak at this developmental stage (Fig. 5). Malate metabolism has been a strong focus for grape berry research, as the malate content of grape juice is a critical determinant for the growth of microbes responsible for wine fermentation. Unlike many other fruits, grapes do not contain a large amount of citrate but accumulate malate as the major organic acid during ripening (Sweetman *et al.*, 2009). Transcriptomic analysis and enzyme activity assays have pointed to an important role for PEPC in malate accumulation during the early phase of grape berry development (Diakou *et al.*, 2000). Indirect evidence for the presence of BTPC-containing Class-2 PEPC complexes in immature grape berries stems from *I*_*50*_(malate) measurements of ~1.2–2 mM determined for total PEPC activity of clarified extracts from unripe grapes (Diakou *et al.*, 2000). This value is comparable to the *I*_*50*_(malate) of 1.6 mM obtained for total PEPC activity present in stage V–VII developing COS extracts that contain approximately equivalent proportions of Class-1 and Class-2 PEPCs (Blonde and Plaxton, 2003). Future studies are needed to assess the possible occurrence of BTPC-containing Class-2 PEPC complexes in immature grape berries and their impact on malate metabolism and overall development of this important crop.

**Table 1.** Significant BTPC expression correlates with elevated intracellular concentrations of L-malate

Species/ Tissue	Developmental stage	Estimated malate concentration (mM)	Reference
Castor endosperm	stage III*	80	Wheeler et al., 2016
	stage IV*	80	
	stage V*	80	
	stages VI-VIII	80	
	stages IX-X	30	
Cotton embryo	5 DPA	50	Li *et al.*, 2010
	10 DPA*	70	
	15 DPA	35	
	20 DPA	20	
	25 DPA	10	
Soybean embryo	stage R5.5*	60	Wheeler *et al.*, 2016
	stage R6	50	
	stage R6.5	25	
	stage R7	20	
	stage R8	20	
Tomato endocarp	10 DPA (immature green)	11–15	Guillet *et al.*, 2002
	20 DPA (immature green)*	13–23	Mounet *et al.*, 2007
	35 DPA (mature green)*	30–35	
	45 DPA (red ripe)	5–14	
Cucumber endocarp	<11 DAP*	19	Handley *et al.*, 1983
	11 DAP	15	
	23 DAP	15	
	24–44 DAP	7.5	
	45 DAP	11	
Grape berry	Pre-ripe, -30 d	100	Diakou *et al.*, 1997
	Pre-ripe, -5 d*	150	
	Ripe, 0 d	93	
	Post-ripe, +10 d	63	
	Post-ripe, +20 d	37	

Developmental stages having maximal *BTPC* expression (Figs 3 and 5) are indicated with bold font and an asterisk. Intracellular malate concentrations were estimated from data provided in the cited publications by assuming that 1 gFW is equivalent to 0.1 gDW and that 1 gFW is equivalent to 1 mL of aqueous volume. Castor and soybean seed developmental stages are as previously described (Blonde and Plaxton, 2003; Wheeler *et al.*, 2016).

The acidic properties of tomato fruit are caused by high concentrations of malate and citrate, which are used to maintain turgor for rapid fruit expansion (Guillet *et al.*, 2002). Tomato fruit malate levels begin to rise at about 10 DPA, when they are immature and green, and maximize at about 30 mM at the mature green stage (Table 1) (Guillet *et al.*, 2002, Mounet *et al.*, 2007), during which *BTPC* transcript levels also peak (Fig. 5). Results discussed above and presented in Figs 6–8 confirmed the presence of high molecular mass Class-2 PEPC complexes composed of BTPC subunits and fruit-specific PTPC subunits in extracts of immature tomato fruit. Tomatoes are similar to COS in that they undergo high rates of respiration but likely exhibit restricted atmospheric gas exchange due to their bulky size (Sweetman *et al.*, 2009). The presence of a Class-2 PEPC complex in tomato fruit may therefore reflect its important CO_2_-refixing role to prevent respiratory inhibition by accumulated CO_2_, potentially facilitated by the association of Class-2 PEPC with the outer mitochondrial envelope (Park *et al.*, 2012). Developing cucumbers also accumulate a relatively high level of malate of about 20 mM (Table 1) which is also needed for osmoregulation and turgor maintenance (Handley *et al.*, 1983). The BTPC and PTPC containing Class-2 PEPC complex of developing cucumber fruit (Fig. 7) is hypothesized to have a similar dual function to mediate rapid anaplerotic PEP carboxylation whilst simultaneously refixing respired CO_2_.

BTPC may also have an important metabolic role in the fibre cells of developing cotton seeds, which are amongst the fastest growing cells in the plant kingdom (Li *et al.*, 2010). Cotton fibres are hair-like single cells that undergo a stage of rapid elongation to several centimeters at anthesis. PEPC activity, intracellular malate, and *BTPC* transcripts of elongating cotton fibre cells simultaneously peak between 5–10 DPA, during which the intracellular malate concentration ranges between 50–70 mM (Fig. 5 and Table 1) (Li *et al.*, 2010). High levels of malate are used for osmoregulation by growing fibre cells to maintain turgor, and malate accumulation shows a strong correlation with the rate, extent, and developmental pattern of fibre elongation (Li *et al.*, 2010). Malate was also suggested to support leucoplast fatty acid synthesis for the production of membrane lipids by the fibre-producing cells, which are incorporated into the rapidly expanding plasma membrane and tonoplast (Li *et al.*, 2010).

It is important to emphasize that significant non-floral *BTPC* expression appears to be specific to malate-accumulating sink tissues. For example, orange fruit endocarp does not appear to have any BTPC or Class-2 PEPC present (Perotti *et al.*, 2010), which is supported by the absence of BTPC peptides in tryptic digests of the orange endocarp proteome (Katz *et al.*, 2007). The early stages of orange fruit development are dominated by quinate and oxalate, followed by a huge increase in citrate (Albertini *et al.*, 2006). Interestingly, a heterologously-expressed 107 kDa PTPC derived from orange fruit endocarp formed an unusual homohexameric native structure that was desensitized to malate inhibition, exhibiting an unusually high *I*_*50*_(malate) value of about 5 mM (Perotti *et al.*, 2010). Although similar to C_4_ and CAM photosynthetic Class-1 PEPCs (Izui *et al.*, 2004), this *I*_*50*_(malate) value is well beyond the range of most Class-1 PEPCs from C_3_ plant sources (O’Leary *et al.*, 2011*b*) and indicates that certain C_3_ species have evolved atypical, malate-desensitized PTPC isozymes. Organic acid accumulation in the fruits of lemons and limes also follow a similar trend, namely high levels of quinate early in development, followed by a large increase in citrate, which suggests that citrus fruits in general may not require BTPC-containing Class-2 PEPCs. Similarly, other fruits such as kiwi, mango, and strawberry rely less on malate and more on the hydrolysis of stored starch as their carbon source during ripening (Sweetman *et al.*, 2009). Indeed, *BTPC* transcripts were essentially absent in developing strawberries (Fig. 4), providing indirect evidence in support for a role of BTPC/Class-2 PEPC in growing sinks that specifically accumulate high malate concentrations.

### Concluding remarks

Owing to the critical position of PEPC and its multifaceted functions in central plant metabolism, it is perhaps not unexpected that an intricate and unprecedented variety of post-translational control mechanisms have evolved to regulate its activity. Regulatory phosphorylation and monoubiquitination, along with variations in cytosolic pH and allosteric effector levels have all been described as strategies to control Class-1 PEPCs in different plant tissues (O’Leary *et al.*, 2011*b*). The discovery in green microalgae and then developing COS of unusual, allosterically desensitized Class-2 PEPC heteromeric complexes composed of tightly interacting but distantly related PTPC and BTPC subunits, added another layer of complexity to the physiological roles and metabolic control of this important CO_2_-fixing plant enzyme. Results of the present study represent an important advance since they demonstrate that: (i) significant non-floral *BTPC* expression is not unique to castor plants but is prevalent in various biosynthetically active, malate-accumulating sink tissues of diverse dicot species, and (ii) *BTPC* transcripts of immature tomato and cucumber fruit are translated into 118 kDa BTPC polypeptides that tightly interact with endogenous PTPC polypeptides to form a Class-2 PEPC. Assessing the potential involvement of Class-2 PEPC and reversible BTPC and PTPC phosphorylation in anaplerotic PEP carboxylation, malate metabolism, and respiratory CO_2_ refixation in these agronomically important tissues will be an important avenue for future research. A major disadvantage of castor is that although its genome has been sequenced and annotated, efficient castor transformation protocols have yet to be developed. This makes it problematic to test the function of BTPC and Class-2 PEPC in COS development and carbon metabolism via gene silencing. By contrast, tomato transformation protocols are well established (Arshad *et al.*, 2014), so it will be of particular interest to assess the impact of RNAi- or CRISPR/Cas9-mediated *BTPC* silencing on tomato fruit development and organic acid metabolism.

## Supplementary Data

Supplementary data are available at *JXB* online.

File S1. Perl script used for RPKM calculations.

Dataset S1. Gene identifiers used for BTPC transcriptome analysis and phylogenetic tree construction.

Dataset S2. *BTPC* expression levels derived from analysis of publicly available RNA-Seq and microarray datasets.

Fig. S1. Alignment of deduced amino acid sequences of BTPCs used for transcriptomic and/or phylogenetic analysis.

Fig. S2. LC-MS/MS identification of *in vivo* Ser-11 phosphorylation site of 107 kDa PTPC polypeptides co-IP’d from immature tomato fruit.

## Supplementary Material

supplementary Data

## Abbreviations:

anti-BTPC: rabbit antibodies raised against castor bean bacterial-type phosphoenolpyruvate carboxylase
anti-PTPC: rabbit antibodies raised against castor bean plant-type phosphoenolpyruvate carboxylase
BAR: Bio-Analytic Resource for plant biology
BTPC: bacterial-type phosphoenolpyruvate carboxylase
co-IP: co-immunopurification
COS: castor (*Ricinus communis*) oilseed
DAP: days after pollination
DPA: days post-anthesis
FPKM: fragments per kilobase of transcript per million reads
*I_50_* (malate): malate concentration causing 50% inhibition of phosphoenolpyruvate carboxylase activity
MS: mass spectrometry
MS/MS: tandem mass spectrometry
PEP: phosphoenolpyruvate
PEPC: phosphoenolpyruvate carboxylase
PTPC: plant-type phosphoenolpyruvate carboxylase
RcPPC3: PTPC isozyme of developing COS
RcPPC4: BTPC isozyme of developing COS
RPKM: reads per kilobase of transcript per million reads
