# Supplementary material for: Transcript profiling indicates a widespread role for bacterial-type phosphoenolpyruvate carboxylase in malate-accumulating sink tissues
Source: J Exp Bot. 2017 Dec 12;68(21-22):5857–69. doi: 10.1093/jxb/erx399 (PMC5854131; doi:10.1093/jxb/erx399)

Supplementary Fig. S1. Alignment of deduced amino acid sequences of BTPCs used for transcriptomic and/or phylogenetic analysis. The solid boxes with corresponding roman numerals indicate three conserved subdomains essential for PEPC catalytic activity (Izui *et al.*, 2004). The dashed rectangle denotes the intrinsically disordered region which corresponds to residues 325-467 of castor BTPC. The red line at the C-terminus indicates the tetrapeptide (R/K)NTG characteristic of non-archaeal, prokaryotic-like PEPCs (O'Leary *et al., 2011b*).

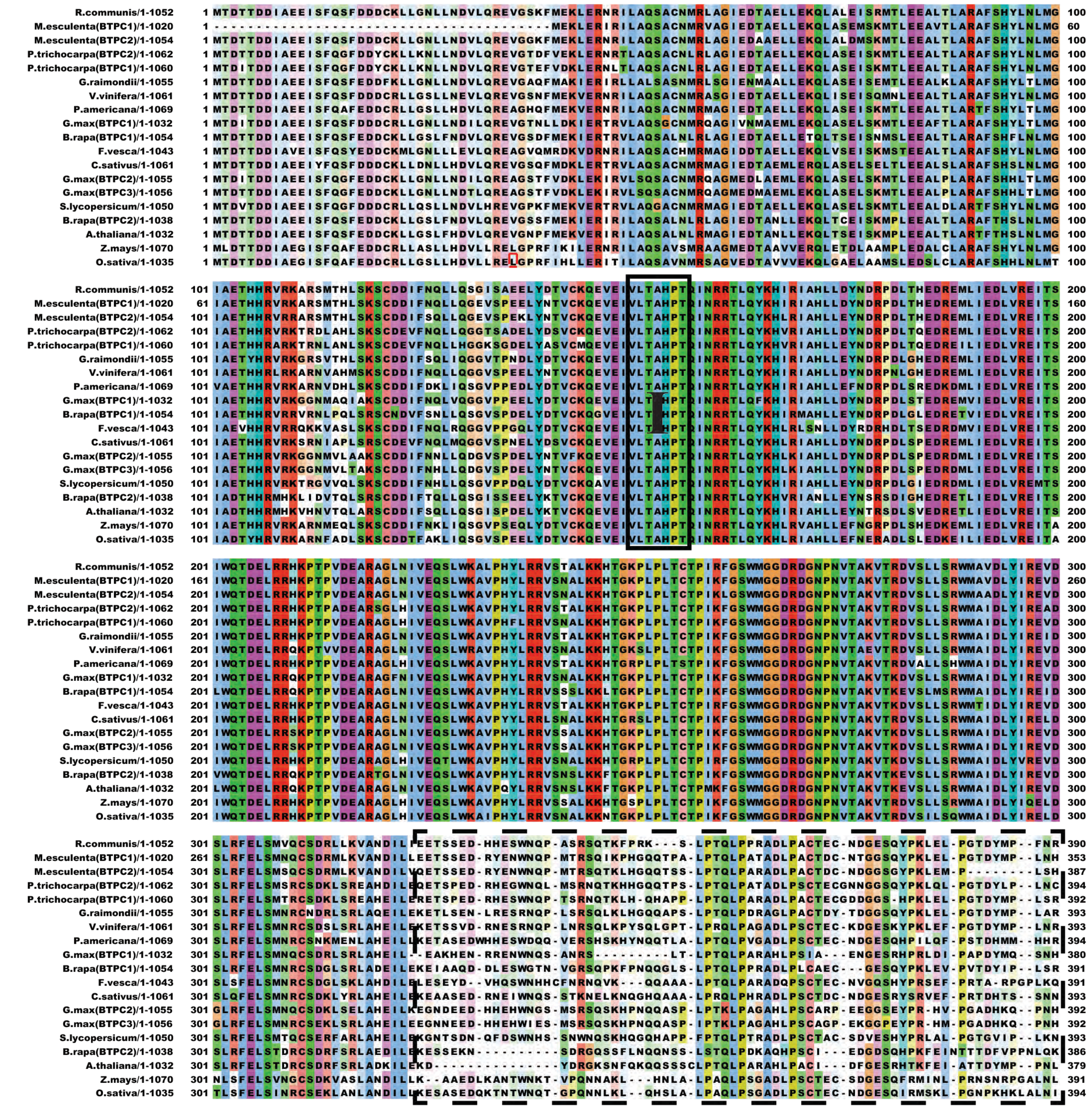

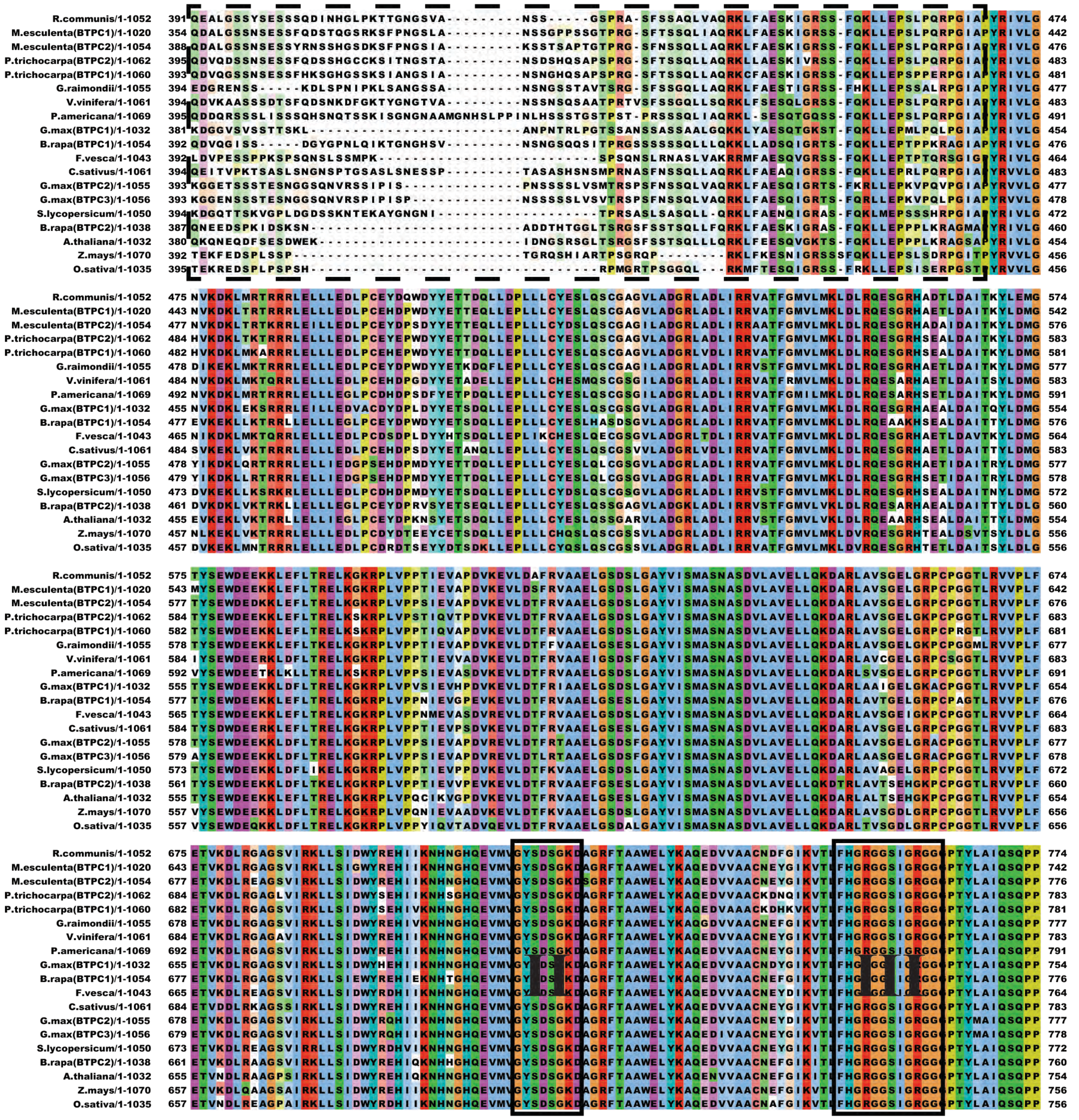
R.communis/1-1052
M.esculenta(BTPC1)/1-1020
M.esculenta(BTPC2)/1-1054
P.trichocarpa(BTPC2)/1-1062
P.trichocarpa(BTPC1)/1-1060
G.raimondii/1-1055
V.vinifera/1-1061
P.americana/1-1069
G.max(BTPC1)/1-1032
B.rapa(BTPC1)/1-1054
F.vesca/1-1043
C.sativus/1-1061
G.max(BTPC2)/1-1055
G.max(BTPC3)/1-1056
S.lycopersicum/1-1050
B.rapa(BTPC2)/1-1038
A.thaliana/1-1032
Z.mays/1-1070
O.sativa/1-1035
II
III

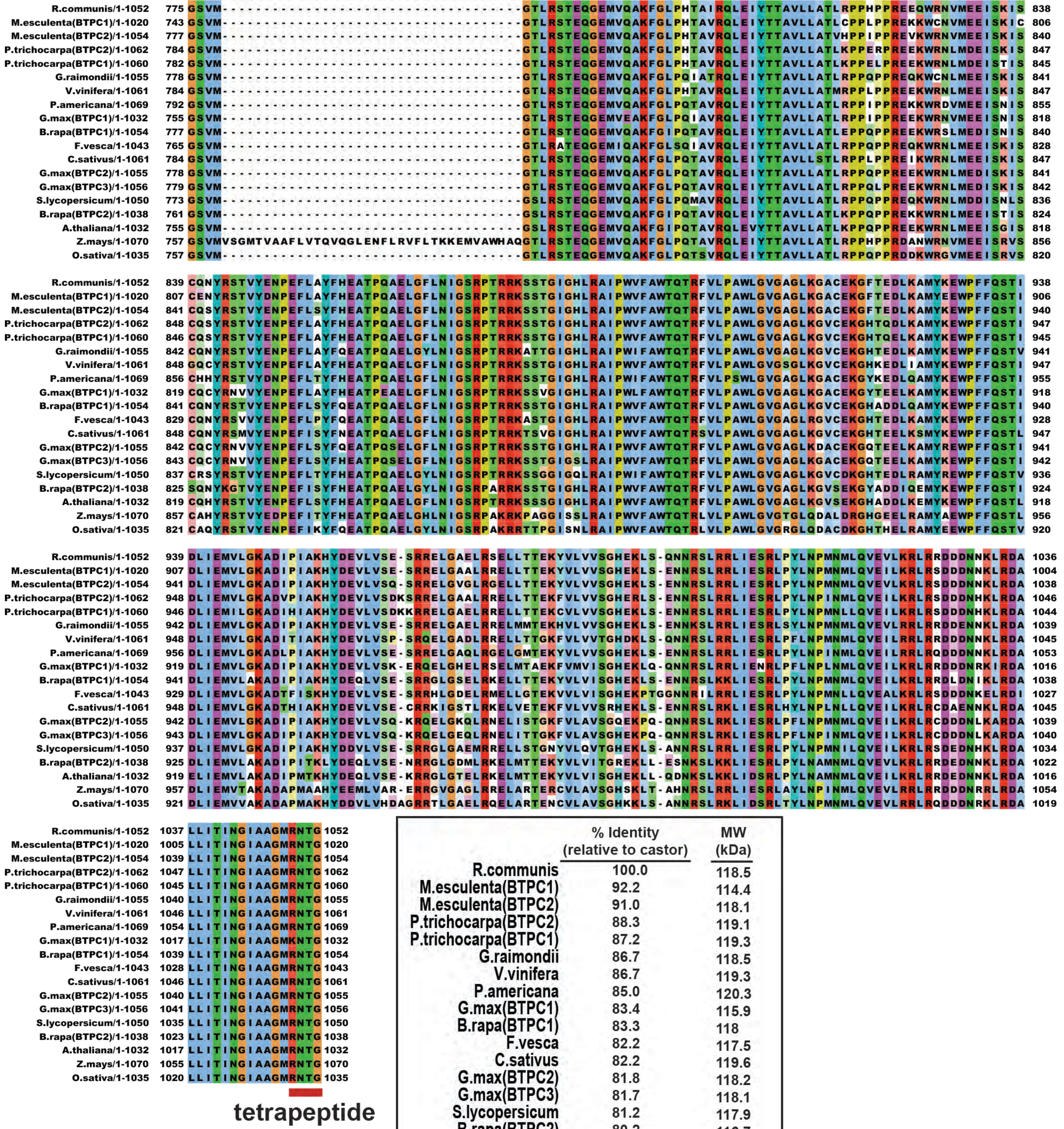

	% Identity (relative to castor)	MW (kDa)
R.communis	100.0	118.5
M.esculenta(BTPC1)	92.2	114.4
M.esculenta(BTPC2)	91.0	118.1
P.trichocarpa(BTPC2)	88.3	119.1
P.trichocarpa(BTPC1)	87.2	119.3
G.raimondii	86.7	118.5
V.vinifera	86.7	119.3
P.americana	85.0	120.3
G.max(BTPC1)	83.4	115.9
B.rapa(BTPC1)	83.3	118
F.vesca	82.2	117.5
C.sativus	82.2	119.6
G.max(BTPC2)	81.8	118.2
G.max(BTPC3)	81.7	118.1
S.lycopersicum	81.2	117.9
B.rapa(BTPC2)	80.2	116.7
A.thaliana	80.0	116.6
Z.mays	78.1	119.4
O.sativa	77.6	115.9

Supplementary Fig. S2. LC-MS/MS identification of *in vivo* Ser-11 phosphorylation site of 107 kDa PTPC polypeptides co-IP'd from immature tomato fruit. (A) The phosphopeptide of *m/z* 559.7391 (2+) was identified by MS/MS measurements at residues 9-17. The collision-induced dissociation fragmentation of the peptide at *m/z* 801.87 yielded the characteristic phosphopeptide fragments with the addition or the neutral losses of 80 Da (HPO_3), 18 Da (H_2O) and 98 Da (H_3PO_4) starting the N-terminal fragment b_3 and the C-terminal fragment y_7, indicating the phosphorylated residue at Ser-11. (B) The collision-induced dissociation of the peptide of *m/z* 801.8711 (2+) at residues 5-17 resulted in a series of relatively high abundance fragments with the addition of 80 Da (HPO_3), further supporting the identity of a phosphorylation site at Ser-11.

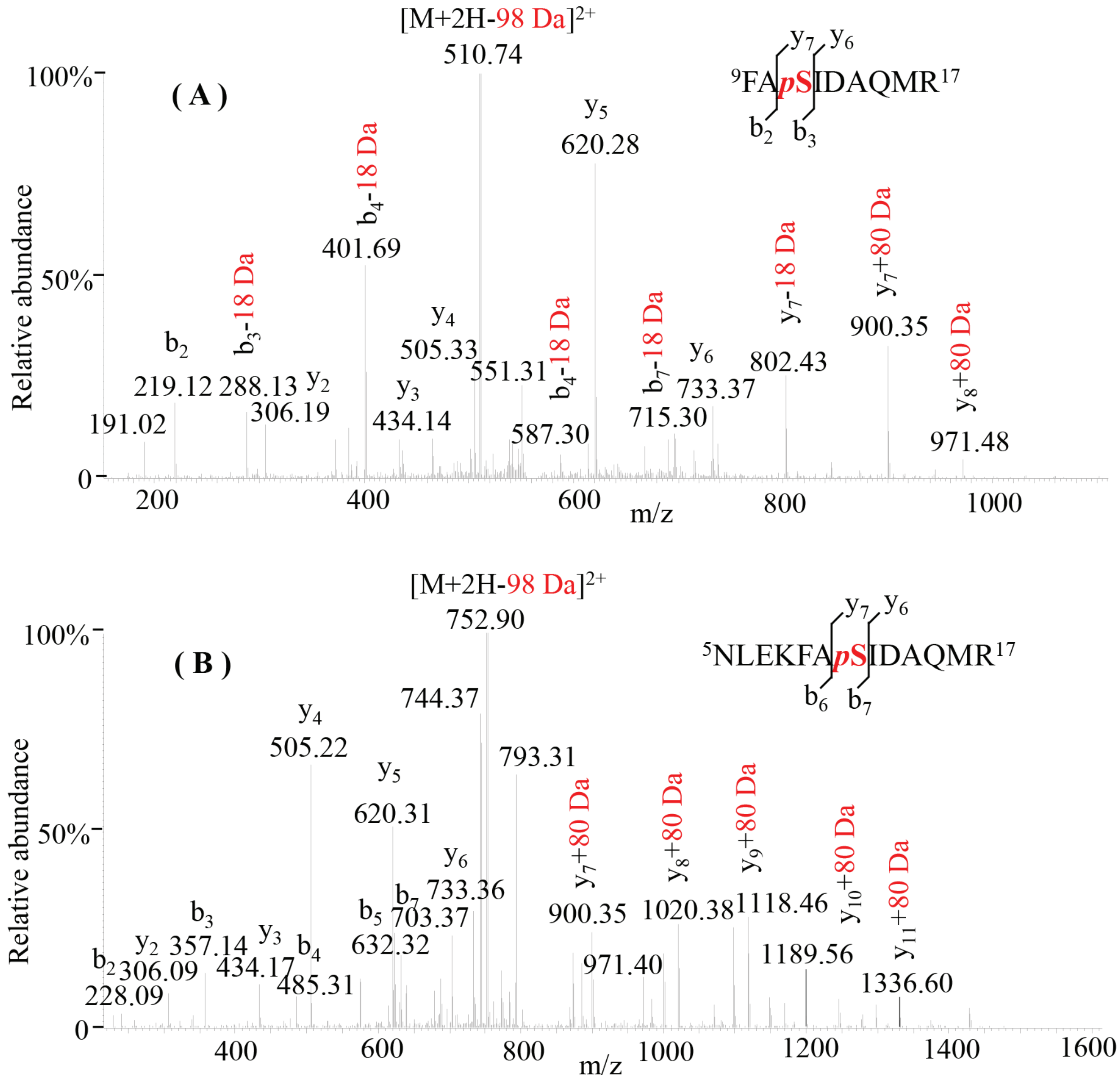

Supplement: supplementary Data [file erx399_suppl_supplementary-data.zip › erx399_suppl_supplementary-figures-S1-S2.pdf]
